# Cranial cooling during firefighting recovery effects on physiological and perceptual strain

**DOI:** 10.1186/2046-7648-4-S1-A1

**Published:** 2015-09-14

**Authors:** Stephen S Cheung, Phillip J Wallace, Anais Masbou, Stewart R Petersen

**Affiliations:** 1 Environmental Ergonomics Laboratory, Department of Kinesiology, Brock University, Ontario, Canada; 2 Faculty of Physical Education and Recreation, University of Alberta, Canada

## Introduction

We performed a firefighting simulation of repeated work-rest bouts while wearing fire-fighting ensemble (FFE) and self-contained breathing apparatus (SCBA) in the heat, removing the helmet and SCBA during recovery and comparing cranial cooling (CC) versus passive (CON) exposure. We hypothesized that CC would better counteract heat storage compared to passive cooling.

## Methods

Eleven males (mean (SD), 30.9 (9.2) y, 49.5 (5.1) mL.kg^-1^.min^-1 ^$\overset{\cdot }{\text{V}}{\text{O}}_{2peak}$) performed two trials consisting of 2 × 20 min bouts of treadmill walking (5.6 km.h^-1^, 4 % incline) in 35 °C and 60 % relative humidity while wearing full FFE and SCBA, with 20 min passive recovery between each exercise bout. During recovery, participants sat in the chamber and removed gloves, helmet, fire hood, and SCBA, but the jacket remained buttoned up. For CC, a close-fitting custom liquid-perfused hood pumped 13 °C water at a rate of ~500 mL.min^-1 ^through the head and neck regions. For CON, participants performed the same recovery but the hood was not perfused to simulate wearing the fire hood and helmet. Rectal temperature (T_re_), heart rate (HR), and ventilation (${\overset{\cdot }{\text{V}}}_{\text{E}}$) were continuously recorded throughout exercise and recovery, while subjective ratings of perceived exertion (RPE), thermal comfort (TC), and breathing stress (BrS) were obtained every 4-5 min during exercise and recovery. Significance was set at p = 0.05.

## Results

All participants successfully completed the first exercise bout, with no differences in any variable prior to the experimental recovery manipulation. Rectal temperature rose in both CC (0.11 ± 0.19 °C,) and CON (0.26 ± 0.15 °C) during Rest, with non-significant interaction between conditions (p = 0.076). During Rest, neck temperature was lower in CC compared to CON from 4 min (CC: 35.73 ± 3.28 °C, CON: 37.66 ± 1.35 °C, p = 0.025) until the end (CC: 33.06 ± 4.70 °C, CON: 36.85 ± 1.63 °C, p = 0.014). HR significantly decreased over recovery in both CC and CON, with no significant differences between conditions. Perceptually, TC was significantly lower in CC at 5 min, 10 min, 15 min, and 20 min time points during the Rest period compared to CON. There were no significant differences in tolerance times (voluntary termination or T_re _= 40 °C) between CC (16.55 (1.14) min) and the CON (16.60 (1.31) min). T_re _was not significantly different at the start (CC: 38.3 (0.40) °C, CON: 38.40 (0.16) °C) and at the end (CC: 38.82 (0.23) °C, CON: 39.07 (0.22) °C) of Exercise2. HR was not significantly different at the start (CC: 149 (17.6) b.min^-1^, CON: 157 (15.6) b.min^-1^) and at the end (CC: 162 (18.76) b.min^-1^, CON: 174 (12.13) b.min^-1^) of Exercise2. Total V_E _during Exercise2 was similar between CC (1146.3 (331.9) L) and CON (1173.3 (307.0) L) as was BrS and RPE.

## Discussion

The face and head has a high alliesthesial thermosensitivity [1], but Tyler *et al*. proposed [2] that a sufficient thermal strain threshold was necessary for neck cooling to be effective. However, our high rate of heat stress potentially overwhelmed any cooling benefit from CC, as any attenuation in physiological and perceptual responses was transient. Thermal hyperpnea was also not alleviated by CC, with no effect on ventilatory demands and air usage. While head and neck cooling during recovery may attenuate thermal discomfort, it is not an effective strategy to decrease the rate of physiological strain or extend tolerance time during heavy exercise in the heat when recovery is performed while largely encapsulated.

## References

[B1] Tyler CJ Wild P Sunderland C Practical neck cooling and time-trial running performance in a hot environment Eur J Appl Physiol 2010 110 1063 1074 10.1007/s00421-010-1567-7 20694731

[B2] Cotter JD Taylor NAS The distribution of cutaneous sudomotor and alliesthesial thermosensitivity in mildly heat-stressed humans: An open-loop approach J Physiol 2005 565 335 345 10.1113/jphysiol.2004.081562 15760945 PMC1464483

